# Vitamin D status in under five children in diverse communities of Karachi

**DOI:** 10.12669/pjms.35.2.680

**Published:** 2019

**Authors:** Khemchand N Moorani, Muhammad Ayaz Mustufa, Syed Furqan Hasan, Naseem Kubar

**Affiliations:** 1 *Khemchand N Moorani, FCPS, MCPS, MBBS. Professor of Pediatric Nephrology, Department of Pediatric Nephrology and Pediatric Medicine Unit-III, National Institute of Child Health (NICH), Jinnah Sindh Medical University (JSMU), Karachi, Pakistan*; 2 *Muhammad Ayaz Mustufa, PhD, MBE, M Phil, CRCP. Director, Sindh Health Care Commission/Senior Research Officer, Pakistan Health Research Council*; 3 *Syed Furqan Hasan, MBBS, MPhil. Associate Professor (Microbiology), Department of Pathology, National Institute of Child Health (NICH), Jinnah Sindh Medical University (JSMU), Karachi, Pakistan*; 4 *Naseem Kubar, MBBS, FCPS trainee. Department of Pediatric Medicine, Unit-III, National Institute of Child Health (NICH), Karachi, Pakistan*

**Keywords:** Vitamin D status, Zinc Levels, Nutritional Status, Sun light exposure

## Abstract

**Background & Objective:**

Vitamin D deficiency (VDD) prevalence is very high in pediatric population in developing countries including Pakistan. VDD contribute significantly to morbidity and mortality among children under five years. Therefore, it is vital to study vitamin D levels in population for future interventions and disease control. Our objective was to determine 25(OH)D levels in children of one to 59 months of age in socio-economically diverse communities of Karachi, Pakistan.

**Methods:**

The cross- sectional analytical survey was carried out over 6 months from January -June 2017. Following proportionate sampling technique four clusters were randomly selected from Korangi, Saddar, Sindhi para and Manzoor colony, Karachi. Blood samples for 25(OH)D and zinc levels were carried out using ELISA and colorimetry. VD level <20 ng/ml was defined as VDD and serum zinc <65ug/dl as low zinc levels. Data including area of residency, gender, ethnicity, parent’s education, family income, house status, duration of sun exposure and history of VD and zinc intake in last 3 months was collected from parents / care seekers on pretested and pre-coded semi-structured questionnaire. Data was analyzed using SPSS version-20. Frequencies and percentages were computed for categorical variables like gender, type of malnutrition whereas mean with standard deviation was used for VD and zinc levels and vitamin D status was compared in three different residential categories according to nutritional status.

**Results:**

Out of 120 children, 67 (56%) were boys and 53 (44%) girls. Mean VD level was 22.8±14.8 ng/ml. Around 60% (70) children were VD deficient, whereas 15 % (18) had insufficient 25(OH)D levels. VDD was more prevalent among low socio-economic group with no obvious difference in age category. Mean zinc level was 123.8±47.45 ug/dl and it was either normal or high (42%) rather than low. Malnutrition was observed in 65% children and majority (82%) of them were undernourished. Optimal sun exposure was reported in 24% children only.

**Conclusion:**

Vitamin D deficiency was highly prevalent in our study population. Children of low socio-economic strata and with sub-optimal sun light exposure are at high risk of vitamin D deficiency. Unexpectedly, high zinc levels in majority of our children with low VD status needs further evidence to substantiate this inverse relation.

## INTRODUCTION

Vitamin D plays an important role in body’s immunological functions, nutritional status, physical and cognitive growth of children.^1^ Vitamin D deficiency and VD insufficiency has been found in all age groups including children and pregnant mothers belonging to different socioeconomic strata from all over the World.^2^,^3^

More than one billion individuals including children and adults are pretentious for low levels of VD around the world.^3^,^4^ High prevalence of VDD is reported in developing countries mainly from South Asia.^4^-^6^ Pakistan and India inhabit a larger share of VDD population. The poverty and prevailing malnutrition continue to exacerbate the magnitude of VDD. It is reported that 84% of pregnant women in India and 70% of healthy volunteers in Pakistan are VD deficient.^3^,^6^ VDD has been neglected in Pakistan as an issue of public health significance for several reasons such as low dietary intake of VD, inappropriate exposure to sunlight due to several sociocultural and economic determinants.^5^ The reported cut-off limits for VDD is serum 25(OH)D level < 20 ng/ml and for VD insufficiency as <30ng/ml.^5^

A high prevalence of VDD has been found in various disorders including diabetes, chronic kidney disease and respiratory tract infections.^7^-^9^ Exacerbation of asthma, reactivation of tuberculosis and human immunodeficiency virus (HIV) have also been reported in patients with low VD levels.^10^-^12^

VDD is the most common cause of nutritional rickets and growth failure in children. It has been found that vitamin D receptors (VDR) regulate gene affecting cellular growth. In addition, VDR in combination with zinc affect intracellular pathways of VD dependent genes.^12^,^13^

Assessing the vitamin D status and magnitude of VDD in various pediatric population groups in Pakistan is a demanding task since majority of children with VD deficiency are asymptomatic.^6^,^14^

In the current study, we targeted apparently healthy children under five years of age to determine the vitamin D status from diverse communities of Karachi with different socio economic and cultural background.

## METHODS

This was a cross- sectional study, conducted in four clusters of Karachi over the period of 6 months from January -June 2017. Study cluster sites were categorized based on monthly income and basic infrastructure. These sites were Sindhi Para as very low socioeconomic (VLSE), Korangi as low (LSE), Manzoor colony as lower middle (LMSE) and Saddar as an upper middle economic (UMSE) stratum.

World Health Organization (WHO) sampling calculator was used with addition of 10% for non-compliance. A total 120 children registered through non-probability, proportionate sampling.

After approval of funding, ethical clearance was obtained from Institutional Ethical Review Board of National Institute of Child Health, Karachi (IERB No. 12/2016) to conduct the study. Informed written consent was obtained from parents / care givers of potential participants after explaining purpose of the study.

Four diverse socioeconomic clusters as described above of respective community through simple balloting were identified. In each cluster, at-least 100 households identified for screening for VD in children of one month to 59 months of age. Children who received vitamin D in last 3 months or on vitamin D supplementation were excluded.

Enrolled children were assessed by physician using anthropomesurments. Data was collected on pre-coded semi structured questionnaire from parents / care givers of children like anthropomesurments including BMI, age, gender, socioeconomic status, parental level of education and duration of sun exposure (hours) per day during last three months. Sunlight exposure less than 30 minutes per day was taken as suboptimal exposure.

Nutritional status was defined as per WHO growth standard charts. Children were categorized as underweight (Z-score < −2), normal weight (Z-score ≥ −2 and ≤1), overweight (Z-score > 1 and ≤ 2), or obese (Z-score > 2).^15^

Blood samples were collected and analyzed at NICH laboratory for 25(OH)D level by ELIZA. Vitamin D status was defined as normal if 25(OH)D level ≥ 30 ng/ml; insufficiency 20 - 29 ng/ml; deficiency 10 - 19 ng/ml and severe VD deficiency if 25(OH)D level was ≤ 10 ng/ml.^16^,^17^

Zinc levels were measured by colorimetry following manufacturer’s guidelines and zinc status was defined as low if it was ≤ 65 ug/dl, normal if between 65-130 ug/dl and high if level was ≥130 ug/dl. The results of each participant were communicated and free of cost consultation was offered.

Data was analyzed using statistical package for social sciences SPSS 20. Frequencies and percentages were computed for categorical variables like gender, type of malnutrition, zinc levels and vitamin D status. Effect modifiers like age, gender, monthly income, parental education, duration of sun exposure was controlled by stratification to see the effect of these variables on outcomes.

## RESULTS

Out of 120 children, 67 (56%) were boys and 53 (44%) girls. We found that 40% were Urdu speaking and 31% were Punjabis. More than 65% families earned less than 20,000/- Pakistani rupees (200 USD approx.) per month. Educational status of both parents was very low with only 12.5% fathers and 10% mothers achieved graduation level. Based on BMI percentile for age, more than 54% children were underweight, 11% overweight and 34% were normal weight. (Table-I)

**Table-I:** Socio demography and nutritional status of study population.

Variable	Frequency	Percentage
***Gender***		
Male	67	55.8
Female	53	44.2
Age in months, mean± S.D (Range) 37.13 17.42 (5 - 60)		
***Ethnicity***		
Urdu	48	40.0
Punjabi	37	30.8
Sindhi	19	15.8
Pashto	8	6.7
Balochi	3	2.5
Others	5	4.2
***Education of Father***		
None	19	15.9
Primary	7	5.8
Middle	25	20.8
Matric	36	30.0
Inter	18	15
Graduate	9	7.5
Post graduate	6	5
***Education of Mother***		
None	22	18.4
Primary	12	10.0
Middle	21	17.5
Matric	34	28.3
Inter	19	15.8
Graduate	12	10
***Family Income***		
Up to 10000	13	10.8
> 10001-20000	66	55.0
> 20001-30000	27	22.5
> 30000	14	11.8
***House Status***		
Kacha	6	5.0
Pacca	91	75.8
Kacha &pacca	18	15.0
Tent	5	4.2
***Nutritional Status of Children***		
Normal weight	41	34.17
Under weight	65	54.17
Over Weight	14	11.66

There were nearly equal number of children were enrolled from each socioeconomic stratum except parents of three children who refused for blood testing, so we have 25 children in VLSE stratum as shown in Table-II. Therefore, 117 children were subjected for 25-OHD and zinc analysis. Mean±SD of vitamin D and zinc level in our study population was 22.8±14.8 ng/ml (min 6.3, max 81) and 123.8±47.45 ug/dl (min 35, max 375) respectively.

**Table-II:** Vitamin D Status According to the Nutritional Status of Children and Socioeconomic Strata N=117.

Socioeconomic Strata (SE)	Nutritional Status	Vitamin D Status				Total (%)

		Normal	Insufficiency	Deficiency	Severe Deficiency	
Low N=29	N	5	3	5	1	14
	U	4	1	3	0	8
	O	3	1	2	1	7
	Total	12	5	10	2	29(24.78)
Lower middle N=31	N	3	2	6	2	13
	U	8	3	3	0	14
	O	1	0	3	0	4
	Total	12	5	12	2	31(26.5)
Upper middle N=32	N	1	0	5	2	8
	U	4	5	10	4	23
	O	0	1	0	0	1
	Total	5	6	15	6	32(27.35)
Very low N=25	N	0	0	4	1	5
	U	0	2	16	1	19
	O	0	0	1	0	1
	Total	0	2	21	2	25(21.36)
Overall status	N	9	5	20	6	40 (34.2)
	U	16	11	32	5	64 (54.7)
	O	4	2	6	1	13 (11.1)
	Total (%)	29(25)	18(15.4)	58(49.6)	12(10)	117(100)

N=normal weight (Z-score ≥ −2 and -<1) U=underweight (Z-score < −2),

O= overweight (Z-score > 1 and ≤ 2).

**Fig.1:**
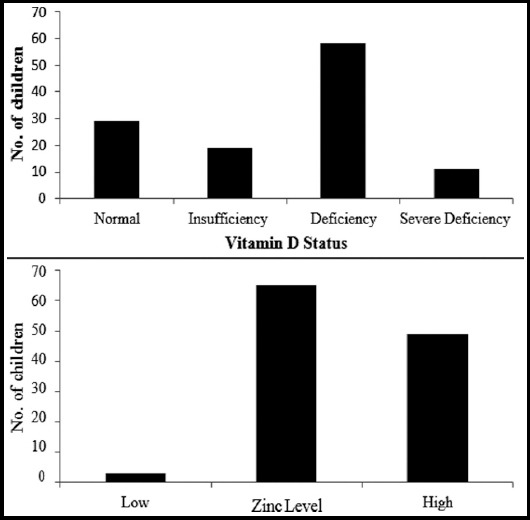
Vitamin D status and Zinc levels in Study Population.

**Fig.2:**
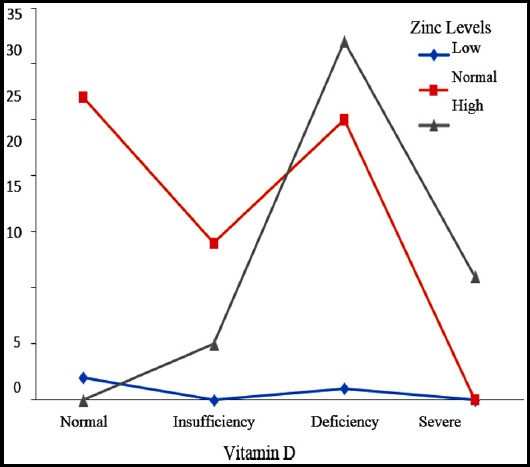
Zinc levels and vitamin D status.

Vitamin D status showed that 60% (70) children were either severe or mild to moderately VD deficient, whereas, 15.4% (18) had insufficient VD levels (Table-II and Fig.1). Zinc levels were high in 42% children and more than 55% children had normal zinc levels (Fig.2). More than 75% children reported for sub-optimal sun exposure whereas optimal sun exposure was observed in only 24% children.

Considering the SE diversity of community, a high frequency of VDD was found among low socio-economic group with no obvious difference in age groups and gender distribution (Table-II). Underweight was highly prevalent in children of all SE strata but more so in VLSE (Sindhi Para 76%) and UMSE cluster (Saddar 71%) whereas 24% from LSE cluster (Korangi) were overweight. Over all 40 children (34%) had normal nutritional status and among them 42% from LMSE, 35% from LSE and 20% from each VLSE and UMSE clusters (Table-II).

Normal zinc level was found in 55% of study population and high zinc levels in 42% of children. All children with high zinc levels had either VD deficiency or insufficiency (Fig.2).

## DISCUSSION

Our study revealed a high prevalence of VD deficiency and insufficiency (75%) in children under five years of age from varied socio-economic strata of Karachi. This could be due to number of factors including suboptimal ultraviolet (UV) light exposure, use of low VD containing food, poor nutritional status of children and low socioeconomic status of families.

Vitamin D is important not only for bone health in children and adults, but also essential for reducing risk of many chronic diseases including cardiovascular, chronic kidney disease, cystic fibrosis, autoimmune diseases and cancers.^8^,^10^,^11^

Reported prevalence of VDD in various studies varies from 70 - 97 % depending upon study design, site and number of participants.^5^,^18^-^21^ Consistent to our finding, most of data collected from community showed similar prevalence of VDD, while higher frequency has been documented in studies mainly conducted in health care facilities with specific disorders.^9^,^14^,^19^,^21^ Therefore, the difference between community-based data and health care facility based generated data need to be segregated for facts accordingly. A high prevalence of VDD in children with growing pain (90%) and chronic kidney diseases (88%) has been reported from tertiary care centers of Karachi.^9^,^14^ Similarly, health care facility-based studies from Karachi on pregnant mothers and their new born showed that 46% of mothers and 88% newborns were vitamin D deficient and it was concluded that maternal vitamin D was significantly dependent on sun exposure and diet.^18^,^19^

We found that all children with sub-optimal sunlight exposure (<30 min) had low VD levels; either insufficiency or deficiency compared to children with optimal sunlight exposure in which all had normal VD status consistent with published work.^1^,^18^-^21^

Almost all the children (100%) from very low socioeconomic area (Sindhi Para) had VDD and VDI suggesting either low VD content in diet or consumption of non-fortified and non-animal source food along with poor exposure to sunlight in peak hours of a day when UV beta rays are optimal for cutaneous VD synthesis. These findings are compatible with a general view point.^1^,^20^-^22^ Furthermore, the co-existence of a high frequency of malnutrition (66%) may also be contributing factor for high magnitude of this micronutrient deficiency in low socioeconomic locality.

Similarly, a very high prevalence of VDD and insufficiency (>60%) in children from lower middle-income groups (Korangi and Manzoor colony) also reflects the less VD intake and suboptimal sunlight exposure.

Surprisingly, a relative high prevalence (85%) of VDD and insufficiency in our upper middle cluster (Saddar) may be explained on densely populated high rise multistory residential area with minimal access to sunlight which is also similar to a recent study in a same age group (under five) children from slum areas of megacity from India.^20^

Many studies have shown micronutrient deficiencies such as iron, zinc and vitamins particularly vitamin A and VDD are common in children with malnutrition.^3^,^6^,^17^, ^21^ Zinc deficiency has been reported in 54.2% children with stunting and supplementation has been recommended in children with diarrhea and acute respiratory tract infection.^23^,^24^ We found normal zinc levels in 55% and surprisingly high zinc levels in 42% instead of low levels. All children with high zinc levels had either VDI or VDD. High zinc levels in our study may be result of modulation effect of zinc on the formation of vitamin D receptor and retinoid X-receptor or an interaction of VD and zinc at cellular level; since zinc is an essential trace element which affect growth, development and maintain integrity of immune system.^25^-^27^

The strength of our study includes; data from diverse communities, due to proportionate sampling there is minimal chance of predictability bias. The causal effect relationship of VDD with sun light exposure and malnutrition has been documented in our study population.

Limitations mainly includes; due to small sample size our findings cannot be generalized. Moreover, bone biochemistry, Dexa scan for bone density, other micronutrient assays and dietary evaluation of foods consumed by the study participants was not done.

## CONCLUSIONS

Vitamin D status in our study population was suboptimal and vitamin D deficiency and insufficiency were highly prevalent. Children of low socio-economic strata and sub-optimal sun light exposure are at higher risk of vitamin D deficiency. Unexpectedly, high zinc levels were found among majority of children with low VD levels; further evidence is required to prove this inverse relation. Food fortification, health education regarding complementary diet and establishment of children play areas in public places for sunlight exposure are the major tools to improve vitamin D status for maintenance of normal physical and mental health.
